# Structural Modeling of Protein Interactions by Analogy: Application to PSD-95

**DOI:** 10.1371/journal.pcbi.0020153

**Published:** 2006-11-10

**Authors:** Dmitry Korkin, Fred P Davis, Frank Alber, Tinh Luong, Min-Yi Shen, Vladan Lucic, Mary B Kennedy, Andrej Sali

**Affiliations:** 1 Department of Biopharmaceutical Sciences, University of California San Francisco, San Francisco, California, United States of America; 2 Department of Pharmaceutical Chemistry, University of California San Francisco, San Francisco, California, United States of America; 3 California Institute for Quantitative Biomedical Research, University of California San Francisco, San Francisco, California, United States of America; 4 Division of Biology, California Institute of Technology, Pasadena, California, United States of America; 5 Department of Structural Biology, Max Planck Institute of Biochemistry, Martinsried, Germany; Columbia University, United States of America

## Abstract

We describe comparative patch analysis for modeling the structures of multidomain proteins and protein complexes, and apply it to the PSD-95 protein. Comparative patch analysis is a hybrid of comparative modeling based on a template complex and protein docking, with a greater applicability than comparative modeling and a higher accuracy than docking. It relies on structurally defined interactions of each of the complex components, or their homologs, with any other protein, irrespective of its fold. For each component, its known binding modes with other proteins of any fold are collected and expanded by the known binding modes of its homologs. These modes are then used to restrain conventional molecular docking, resulting in a set of binary domain complexes that are subsequently ranked by geometric complementarity and a statistical potential. The method is evaluated by predicting 20 binary complexes of known structure. It is able to correctly identify the binding mode in 70% of the benchmark complexes compared with 30% for protein docking. We applied comparative patch analysis to model the complex of the third PSD-95, DLG, and ZO-1 (PDZ) domain and the SH3-GK domains in the PSD-95 protein, whose structure is unknown. In the first predicted configuration of the domains, PDZ interacts with SH3, leaving both the GMP-binding site of guanylate kinase (GK) and the C-terminus binding cleft of PDZ accessible, while in the second configuration PDZ interacts with GK, burying both binding sites. We suggest that the two alternate configurations correspond to the different functional forms of PSD-95 and provide a possible structural description for the experimentally observed cooperative folding transitions in PSD-95 and its homologs. More generally, we expect that comparative patch analysis will provide useful spatial restraints for the structural characterization of an increasing number of binary and higher-order protein complexes.

## Synopsis

Protein–protein interactions play a crucial role in many cellular processes. An important step towards a mechanistic description of these processes is a structural characterization of the proteins and their complexes. The authors developed a new approach to modeling the structure of protein complexes and multidomain proteins. The approach, called comparative patch analysis, complements the two currently existing approaches for structural modeling of protein complexes, comparative modeling, and protein docking. It limits the configurations refined by molecular docking to the structurally defined interactions of each of the complex components, or their homologs, with any other protein, irrespective of its fold; the final prediction corresponds to the best-scoring refined configuration. The authors applied comparative patch analysis to predict the structure of the core fragment of PSD-95, a five-domain protein that plays a major role in the postsynaptic density at neuronal synapses. The study suggests two alternate configurations of the core fragment that potentially correspond to the different functional forms of PSD-95. This finding provides a possible structural explanation for the experimentally observed cooperative folding transitions in PSD-95 and its homologs.

## Introduction

Protein–protein interactions play a key role in many cellular processes [1,2]. An important step towards a mechanistic description of these processes is a structural characterization of the proteins and their complexes [3–6]. Currently, there are two computational approaches to predict the structure of a protein complex given the structures of its components, comparative modeling [6–11] and protein–protein docking [12–15].

In the first approach to modelling a target complex, standard comparative modelling or threading methods build a model using the known structure of a homologous complex as a template [7,10]. The applicability of this approach is limited by the currently sparse structural coverage of binary interactions [6]. In the second approach, an atomic model is predicted by protein–protein docking, starting from the structures of the individual subunits without any consideration of homologous interactions [12–16]. This docking is usually achieved by maximizing the shape and physicochemical complementarity of two protein structures, through generating and scoring a large set of possible configurations [13,16]. Experimental information, such as that obtained from NMR chemical shift mapping, residual dipolar couplings, and cross-linking, can also be used to guide protein docking [17–20]. While docking is applicable to any two subunits whose structures are known or modeled, both the sampling of relevant configurations and the discrimination of native-like configurations from the large number of non-native alternatives remain challenging [15].

### Comparative Patch Analysis

**Figure 1:**
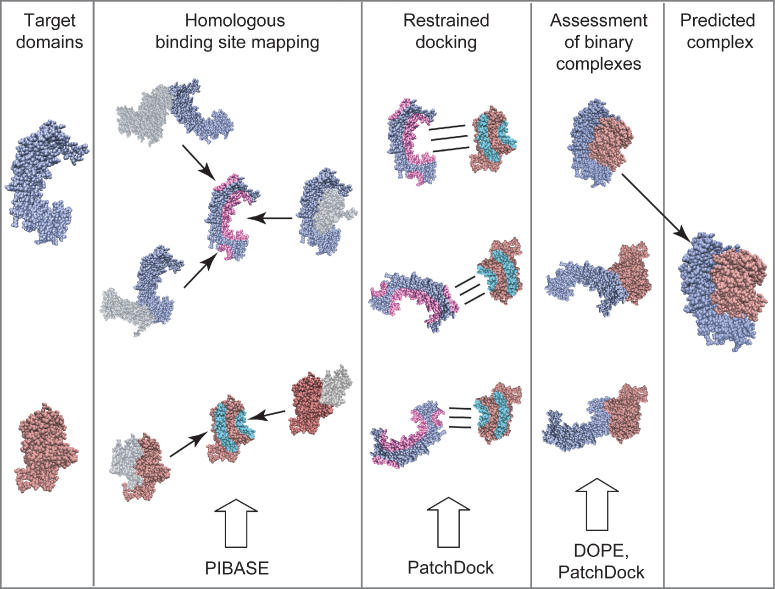
Basic Steps of Comparative Patch Analysis Approach First, the binding sites of the homologs of each domain are extracted from PIBASE and superposed on its surface. Second, for each pair of the superposed binding sites, we apply a restrained docking of the domains with PatchDock to obtain a set of candidate binary domain complexes. Each of the binary complexes is then ranked using geometrical complementarity and statistical potential, and the top-ranked complex is selected to be a final prediction.

Here, we propose a third approach to modeling complexes between two structures (Figure 1). The approach, called comparative patch analysis, is a hybrid of protein docking and comparative modeling based on a template complex, with a greater applicability than comparative modeling and a higher accuracy than docking. Comparative patch analysis relies on our prior analysis of the location of binding sites within families of homologous domains [21]. This analysis indicated that the locations of the binding sites are often conserved irrespective of the folds of their binding partners. The structure of the target complex can thus be modeled by restricting protein docking to only those binding sites that are employed by homologous domains. As a result, comparative patch analysis benefits from knowledge of all interactions involving either one of the two partners.

We find that comparative patch analysis increases the prediction accuracy relative to protein docking. It is able to correctly identify the binding mode in 70% of 20 benchmark complexes, predicting the overall structure with an average improvement in all-atom RMS error of 13.4 Å, compared with protein docking. In contrast, protein docking correctly identifies the binding mode in 30% of the complexes.

### PSD-95 Protein

We apply comparative patch analysis to model the structure of PSD-95 protein. PSD-95 is abundant in the postsynaptic density (PSD), a cytosolic organelle that plays a pivotal role in neuronal signaling [22–26]. PSD-95 serves as a major scaffold for other signaling proteins, participates in receptor and channel clustering, and performs a range of other diverse functions [25–31].

PSD-95 is a member of the membrane associated guanylate kinase (MAGUK) family. It is composed of three PDZ (named after PSD-95, DLG, and ZO-1) domains followed by SH3 (Src homology 3) and GK (guanylate kinase-like) domains [32,33]. Isolated structures of all three PDZ domains as well as the structure of the SH3-GK domain complex have been solved [34–38]. The complete structure of PSD-95 has not been determined, but experiments suggest that it adopts multiple conformations [39,40]. The structures of these conformations are necessary for functional insight into the regulation of PSD-95 activity [40,41].

We apply comparative patch analysis to model the structure of the complex between the third PDZ (PDZ_3_), SH3, and GK domains. These domains comprise 60% of the PSD-95 mass and are the defining domains of the membrane-associated guanylate kinase family. We propose two configurations that satisfied all imposed spatial restraints, including previously observed binding sites, consistency with the given linker length, and physicochemical complementarity of the interacting surfaces. In addition, the prediction is in concordance with and rationalizes available biochemical, structural, and evolutionary data.

The paper begins by comparing the performance of comparative patch analysis with protein docking on a benchmark set of 20 binary protein complexes (Results). Next the application of comparative patch analysis to predicting the structure of PDZ_3_-SH3-GK complex is described (Results). We combine the predictions with existing experimental evidence to propose a mechanism for the intramolecular regulation of PSD-95 (Discussion). In addition, we discuss the advantages and disadvantages of comparative patch analysis and briefly outline future directions. Finally, we present the details of the method (Methods).

## Results

To assess the method, we applied comparative patch analysis to a benchmark set of 20 binary complexes of known structure (Methods). We then used comparative patch analysis to predict the tertiary structure of the PSD-95 core fragment that contains PDZ_3_, SH3, and GK domains.

### Assessment of Comparative Patch Analysis

**Figure 2:**
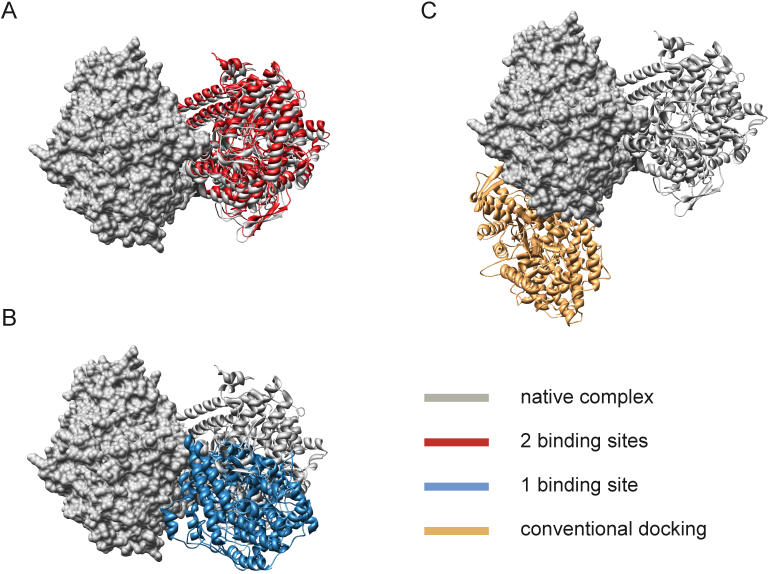
Examples of Predicted Protein Interface between Two Subunits for a Pyruvate Formate–Lyase Protein Complex from Our Benchmark Set Shown are the structures of the native complex (grey) together with the best-scoring models that were predicted by comparative patch analysis using binding site information for (A) both, or (B) just one of the interacting subunits, and (C) by conventional protein docking, where no binding site information is provided. The predicted and native structures are superposed using one of the two subunits, which is represented by its accessible surface. The remaining subunits of the predicted structures are shown in the ribbon representation colored red, blue, and orange, correspondingly. In both scenarios, comparative patch analysis was significantly more accurate than protein docking. Using both binding sites, comparative patch analysis accurately predicted the protein interaction interface, including the relative orientation of subunits. The accuracy of interface prediction by our approach using only one binding site was significantly reduced, while it was still able to predict the binding sites near their native locations. The conventional protein docking failed to accurately predict either the relative orientation of subunits or the locations of their binding sites.

Comparative patch analysis may be applied to two scenarios where binding site information is available for both or just one of the interacting subunits. We compared their performance to that of protein docking (Methods). In both scenarios, comparative patch analysis was significantly more accurate than protein docking (Figure 2). Using both (one) binding site information, the overall structure was improved for 13 (8) of the 20 complexes, with an average improvement in the all-atom RMS error of 13.4 Å (6.1 Å). The interface coverage increased by 29% (6%), and the binding site coverage by 30% (10%), on average (Table 1). In 15 (8) complexes, comparative patch analysis produced models with all-atom RMS error <3 Å, while protein docking achieved this accuracy for only six complexes. Comparative patch analysis identified the interfaces correctly in 15 (9) complexes, including 8 (7) multidomain proteins and 7 (2) protein complexes, while protein docking achieved this for 7 complexes, including 6 multidomain proteins and 1 protein complex. In those 15 complexes, on average 71% of the predicted residue contacts were observed in the native structures (standard error is 5%). As expected, comparative patch analysis was more accurate using binding site information for both interacting domains compared with using only one.

**Table 1:**
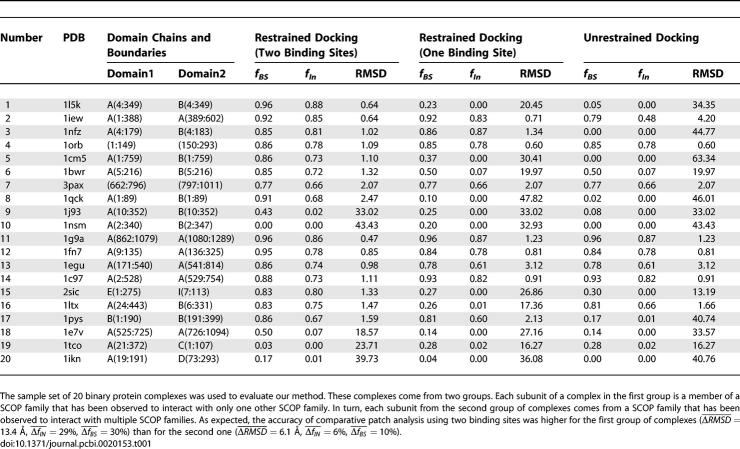
Assessment of Comparative Patch Analysis Approach

### Application to PSD-95

**Figure 3:**
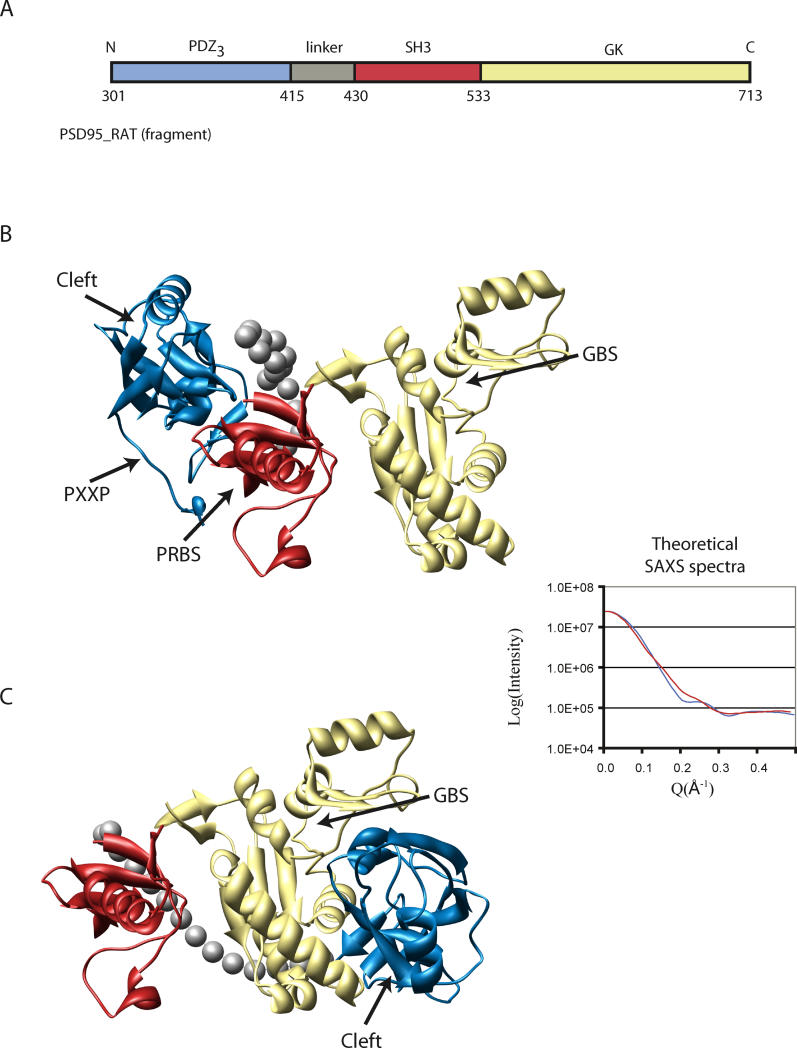
Two Binding Modes of the Core Fragment of Rat PSD-95 The PDZ_3_ domain is shown in blue, SH3 in red, and GK in yellow. The grey spheres correspond to the residues of the interdomain linker between PDZ_3_ and SH3. Locations of the hydrophobic cleft (Cleft) and PXXP motif (PXXP) in PDZ_3_, PRBS in SH3, and the GBS in GK are shown by arrows. (A) The domain architecture of the core fragment. (B) The first predicted configuration. (C) The second predicted configuration. The difference between the theoretically calculated SAXS spectra of the first (red) and second (blue) configurations is significantly larger than the anticipated experimental error.

Next we modeled the tertiary structure of the core fragment of rat PSD-95, which includes the PDZ_3_, SH3, and GK domains (Figure 3, see Figure 3A). As this fragment contains three independent domains, there are three possible domain–domain interactions. The interaction between SH3 and GK domains were known from X-ray crystallography [37,38]. Here we focused on characterizing the other two putative interactions, namely between the PDZ_3_ and SH3 as well as between PDZ_3_ and GK domains. For both cases, we applied comparative patch analysis using two subunits, one containing PDZ_3_ and the second one containing the interacting SH3 and GK domains. The first interaction was modeled using the binding site locations of the PDZ_3_ and SH3 in all known homologs, while the second was modeled using those of the PDZ_3_ and GK homologs (Methods). The results for both interactions are described next, followed by the comparison with results obtained by conventional protein docking.

### PDZ_3_–SH3 Interaction

The comparative patch analysis protocol was applied using the nonredundant sets of 49 PDZ_3_ and 26 SH3 binding sites combined to give all 1,274 possible input pairs. The protocol resulted in an ensemble of 503 models of the PDZ_3_–SH3 complex (Methods).

**Figure 4:**
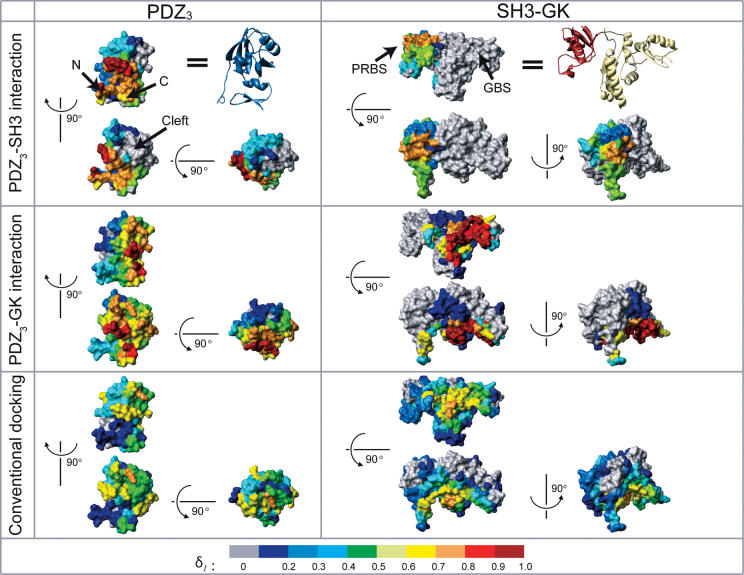
The Localization of Binding Sites for Both Modeled Configurations of the PDZ_3_–SH3–GK Core Fragment Compared with Protein Docking Top ten scoring models were selected for both interactions (PDZ_3_–SH3, PDZ_3_–GK) obtained using comparative patch analysis and using conventional protein docking. The localization index *δ_I_* of a residue defines the relative frequency of its participation in the interaction interface. The residues that are colored grey do not participate in the interface of any of the top ten models. The PRBS in SH3 and the GBS in GK are shown by arrows.

The interface of the best-scoring model (1493 Å^2^) that satisfied the interdomain linker restraint consisted primarily of the C- and N-terminal residues of PDZ_3_ as well as the residues of the proline-rich binding site (PRBS) and the first two beta strands of SH3 (Figure 3B). The PDZ_3_ hydrophobic cleft, known to be essential for binding the C-termini of other proteins, remained accessible in this complex [42,43]. The N-terminus of PDZ_3_ contains a PREP motif (P308, R309, E310, P311) which belongs to the canonical PXXP family of motifs known to interact with the PRBS of SH3 [44–47]. In the best-scoring model, this motif was in proximity to the PRBS (Figure 3B). Our confidence in this predicted binding mode was bolstered when its binding residues were found to occur in regions of high localization derived from the ten best scoring models that satisfied the linker restraint (Figure 4). Ninety-four percent of the binding residues in the best-scoring model were found to occur in no less than 70% of the ten best-scoring models.

### PDZ_3_–GK Interaction

The comparative patch analysis protocol was applied to the PDZ_3_–GK complex using 10,731 input pairs formed by combining the nonredundant sets of 49 PDZ_3_ and 219 GK binding sites. The protocol resulted in an ensemble of 1,929 models (Methods).

The interface of the best-scoring model was extensive (2729 Å^2^), and includes, among others, residues located at the C-terminus and near the hydrophobic cleft of PDZ_3_ as well as a large groove of GK formed by the GMP-binding and LID regions [48–50] (Figure 3C). The analysis of the ten best-scoring models satisfying the interdomain linker restraints revealed high localization of the binding residues for both domains (Figure 4). The residues of PDZ_3_ with the highest localization were located around the domain's hydrophobic cleft and the C-terminus (Figure 4). In addition, the entire GMP-binding site (GBS) of the GK domain and part of the hydrophobic cleft of the PDZ domain became inaccessible in most top-scoring models, including the best-scoring one. Forty-six percent of the binding residues in the best-scoring model were found to occur in no less than 70% of the ten best-scoring models.

### Comparison with Protein Docking Results

To evaluate the effect of binding-site information on modeling the PDZ_3_–SH3–GK complex, conventional protein docking of the PDZ_3_ and SH3–GK domains was performed (Methods). Analysis of the ten best-scoring models satisfying the interdomain linker restraint revealed that the binding sites of both PDZ_3_ and SH3–GK domains were significantly delocalized compared with the comparative patch analysis models. Moreover, the binding residues of the top-scoring models almost completely covered the domain surfaces (93% and 81% of the PDZ_3_ and SH3–GK domains) (Figure 4). The best-scoring model obtained using protein docking was different from both the best PDZ_3_–SH3 and the PDZ_3_–GK comparative patch analysis models (unpublished data).

### PXXP Motif Conservation Analysis

The proximity of the PDZ_3_ PXXP motif and the SH3 PRBS in the predicted model prompted a search for PXXP motifs in the sequences of six PSD-95 proteins and splice variants from four species to assess the significance of this observation. All sequences contained at least one form of a PXXP motif or noncanonical SH3-binding motif that could mimic the PXXP motif (Table 2) [47]. The human, rat, and mouse proteins all contained a PREP motif in PDZ_3_; the zebrafish protein did not. Five other potential SH3 binding motifs were found outside of known domains; two at the N-terminus, one at the C-terminus, and two in the interdomain linker between PDZ_2_ and PDZ_3_. The conservation of the PREP sequence in PDZ_3_ from the mammalian species suggests that its interaction with SH3 may be functionally significant.

**Table 2:**
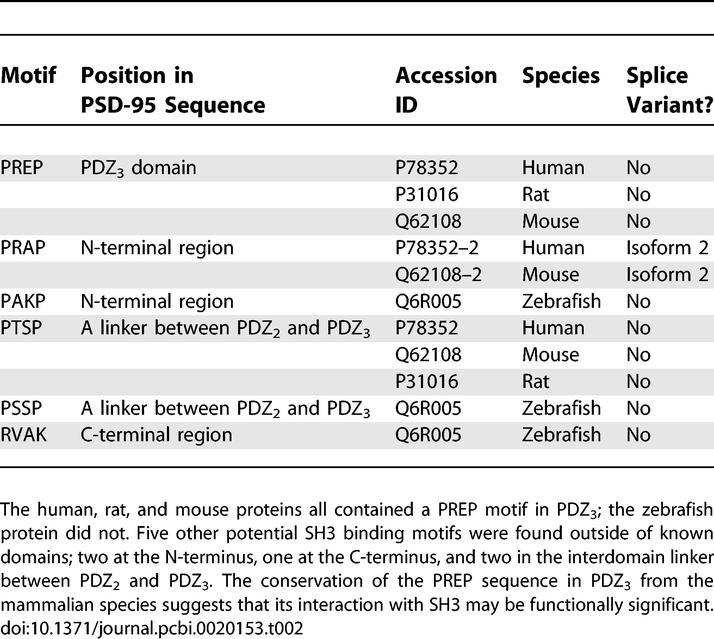
Cross-Species Analysis of PXXP Motif in PSD-95 Proteins

### Proteolysis of PSD-95

**Figure 5:**
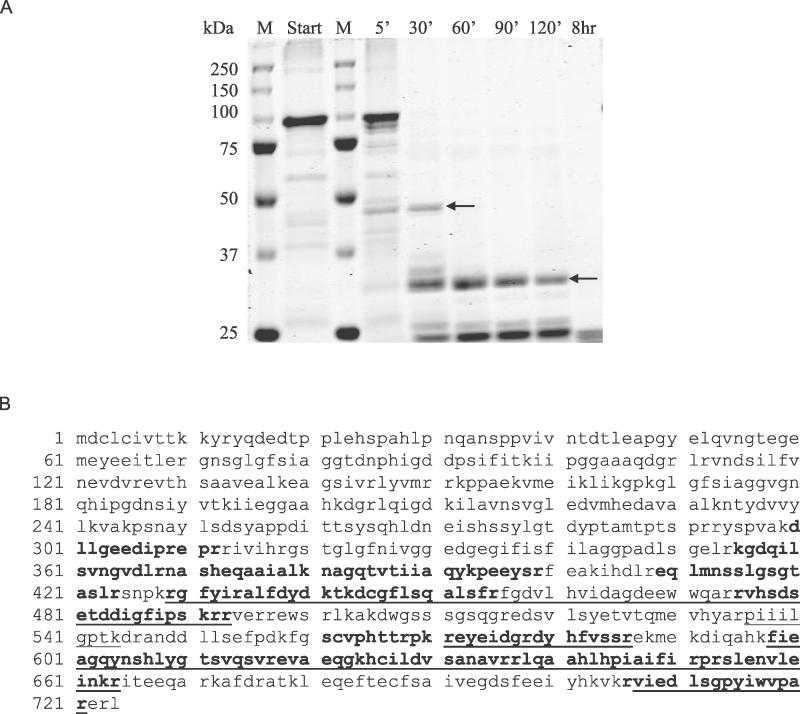
The PDZ_3_–SH3–GK and SH3–GK Domains Are Stable Fragments (A) Coomassie-stained gel (10% acrylamide) of aliquots from limited proteolysis of PSD-95 by Subtilisin proteinase: panels 1 and 3, Precision Plus Protein molecular weight marker (Bio-Rad, http://www.bio-rad.com); panel 2, starting sample prior to proteinase addition; panels 4, Lanes 4–9, Aliquots at 5, 30, 60, 90, 120 min, and 8 h after protease addition (as labeled). Arrows point to stable fragments that were excised from the gel and analyzed by mass spectrometry as described in Methods. (B) Sequence of Rat PSD-95: underlined are the peptide sequences identified by mass spectrometry from the ∼34 kDa stable fragment corresponding to residues 429–721 (33,944 kDa). In bold are the sequences derived from the ∼48 kDa stable fragment comprising residues 300–721 (47,796 kDa).

Limited proteolysis of recombinant PSD-95 with Proteinase K produces a prominent ∼48 kDa band at 30 min (Figure 5). Matrix-assisted laser desorption ionization (MALDI) analysis of peptides generated by tryptic digestion of this band indicates that it represents the sequence from residues 300 to 721, which corresponds to the PDZ_3_ and SH3–GK domains (mass accuracy, Δppm ≤ 13). Further digestion leads to the disappearance of the PDZ_3_–SH3–GK entity and the appearance of a stable ∼34 kDa fragment. The 34-kDa band was identified by MALDI analysis as the SH3–GK domains, encompassing residues 429 to 721 (Δppm ≤ 10 for all detected peptides). Cleavage with thermolysin, another nonspecific protease, generates similarly sized stable fragments (unpublished data).

## Discussion

We have introduced comparative patch analysis, an approach to the modeling of a complex between two subunit structures, and applied it to the protein PSD-95, a key neural-signaling scaffold. The approach relies on structurally defined interactions of each of the complex components, or their homologs, with any other subunit, irrespective of its fold (Figure 1). We assessed comparative patch analysis for its increased applicability relative to comparative modeling as well as increased accuracy relative to conventional protein docking (Figure 2, Table 1). Next, comparative patch analysis was applied to model the structure of a core fragment of rat PSD-95, containing the PDZ_3_, SH3, and GK domains, resulting in two predicted configurations (Figures 3 and 4). The model was experimentally supported by limited proteolysis (Figure 5). In addition, the prediction is in concordance with and rationalizes available biochemical, structural, and evolutionary data (Figures 3 and 4, Table 2).

### Comparative Patch Analysis

By limiting the configurational search to the known binding modes of the homologous subunits and applying a physical assessment of candidate complex structures, comparative patch analysis benefits from the advantages of both homology-driven and physics-driven docking. Its coverage is larger than that of comparative modeling and its accuracy is higher than that of protein docking (Figure 2), although the coverage and accuracy are lower than those of protein docking and comparative modeling, respectively.

At least one binding site is available for 1,989 of the 3,114 total Structural Classification of Proteins (SCOP) domain families (release 1.69, July 2005). Eight hundred fifty of these families contain between ten and 100 binding sites, allowing the exhaustive pairwise docking that is currently required. Thus, the applicability of comparative patch analysis extends to approximately 41%, and in the current implementation is computationally feasible for 8%, of the ∼4,850,000 theoretically possible binary domain–domain interactions. The coverage of conventional protein docking is 100%, while the comparative modeling approach is applicable to only 2,126 pairs of families, which constitutes 0.06% of the theoretically possible interactions.

When compared with protein docking, comparative patch analysis was able to correctly identify the binding mode in 40% more benchmark complexes, predicting the overall structure of the complexes with an average improvement in all-atom RMS error of 13.4 Å. The method also exhibits robustness to small errors in the locations of the specified binding sites, due to the configurational search performed by the docking procedure. In the benchmark set of complexes with known structures, a minimal threshold of 75% overlap between the initially specified and resulting refined binding sites captured all but one of the good models (LRMS error less than 3 Å), while allowing no false positives.

### PSD-95 Protein: Predicting the Structure of the Core Fragment by Analogy

#### Evolutionary and experimental evidence for intermolecular interaction between PDZ_3_ and SH3–GK domains.

When modeling the structure of the PDZ_3_–SH3–GK fragment, we assumed an interaction between the PDZ_3_ and SH–GK domains. PDZ_3_ is a good candidate for interaction with the SH3–GK domains because it is immediately upstream of SH3, separated by a relatively short 14-residue linker. To investigate whether or not PDZ_3_ interacts with SH3–GK, the analysis of domain co-occurrence, as well as limited proteolysis, were applied.

A survey of the domain architectures of proteins that contain both SH3 and GK domains revealed that the proteins either do not have other domains or also contain at least one PDZ domain always preceding the SH3–GK tandem domain. The minimal architecture that contains at least one PDZ, SH3, and GK domain consists of only these three domains. This pattern strongly suggests a physical interaction between the SH3–GK tandem and the preceding PDZ domain [51,52].

The stable fragments resulting from limited proteolysis of PSD-95 by nonspecific proteases reflect the cleavage of accessible loops, rather than cleavage at a particular substrate sequence. We identified stable PDZ_3_–SH3–GK and SH3–GK fragments by mass spectrometry, demonstrating susceptibility of PSD-95 to protease cleavage at sites between the PDZ_2_ and PDZ_3_ domains and between the PDZ_3_ and SH3–GK domains. Limited proteolysis with trypsin (unpublished data) also supports the conclusion that the PDZ_3_ and SH3–GK domains are stable protein structures. These data are consistent with intramolecular interactions between the PDZ_3_ and the SH3–GK domains of PSD-95.

#### Application of comparative patch analysis.

Modeling the structure of the core PSD-95 fragment is challenging for a number of reasons. First, the structures of neither PDZ–SH3 nor PDZ–GK complexes are available, rendering comparative modeling inapplicable in this case. Moreover, conventional protein docking results were ambiguous, generating a varied ensemble of PDZ_3_ and SH3–GK complexes without a predominant binding mode (Figure 3C). On the other hand, each of the domain families is known to repeatedly utilize a small number of binding sites for different protein interactions. For instance, PDZ domains bind the C-termini of several different proteins through its hydrophobic cleft [42,53]. Similarly, the PRBS of SH3 domains recognizes PXXP-sequence motifs in a variety of proteins [45,46]. These observations suggest that comparative patch analysis is suited for modeling the PSD-95 core fragment.

#### Functional roles of the predicted configurations.

Comparative patch analysis of the PDZ_3_–SH3–GK fragment found two possible configurations that satisfied all imposed spatial restraints, including previously observed binding sites, consistency with the given linker length, and physicochemical complementarity of the interacting surfaces. In addition, the ensemble of models produced by comparative patch analysis for each interaction type (PDZ_3_–SH3, PDZ_3_–GK) exhibited a single predominant binding mode. The binding sites forming the interaction interfaces of these models are located at the same or similar regions of the protein surface (Figure 4). Therefore, the binding modes are predicted with relative confidence. Multiple stable configurations of PSD-95 and its close homologs have recently been suggested independently based on biochemical studies [40,54] and single-particle electron microscopy experiments [41]. As we describe below, we suggest the two binding modes have clear functional implications.

The two predicted configurations exhibit structural properties that suggest unique functional roles. In the first configuration, the hydrophobic cleft of the PDZ domain and the GBS of the GK domain are both accessible, suggesting that this configuration corresponds to an active state in which binding of other proteins at these two sites can occur (Figure 3B). These binding sites are thought to mediate intermolecular interactions essential for the scaffolding role of PSD-95 [42,49,55–57]. In contrast, both binding sites are buried in the second configuration, by the interface between the PDZ_3_ and GK domains (Figure 3C), which is suggestive of an alternative functional state. This second configuration points to an efficient intramolecular regulatory mechanism for switching the functional state with a single interaction. Similar regulatory mechanisms have been observed in other signaling networks, such as the TCR and MAPK systems [58,59], indicating this regulation may be a general feature of signaling pathways.

This two-state model also provides a structural explanation for the change in binding affinity between the GK domain and MAP1A protein in the presence of the PDZ_3_ domain [60]. It has been shown that the GK domain alone is able to bind MAP1A. In the presence of PDZ_3_, this binding affinity is dramatically reduced. The affinity is recovered upon titration of a C-terminal peptide of CRIPT known to specifically interact with the hydrophobic cleft of PDZ_3_. This competitive binding suggests that binding to MAP1A and binding to PDZ_3_ are mediated by the same GK binding site. Our model is in complete agreement with this hypothesis and provides a structural explanation for these observations.

It is known that SH3 domains bind proteins with PXXP sequence motifs through their proline-rich binding regions. The proximity of the PDZ_3_ PXXP motif to the SH3 PRBS in the first configuration proposed by comparative patch analysis is consistent with the classical SH3–PXXP motif recognition. A similar PXXP-mediated intermolecular PDZ–SH3 interaction has been previously suggested to occur in syntenin [61]. Sequence analysis of PSD-95 from different species indicates that PXXP motifs are not found in its other two PDZ domains, although such motifs are found in the PDZ_2_–PDZ_3_ linkers and the flexible N-terminus (Table 2). Recent studies have demonstrated the importance of disordered regions in binding events [62], suggesting that future investigation of interactions of these PXXP motifs using recently developed flexible docking algorithms [63] should prove fruitful.

The limited proteolysis experiment (Figure 5) is a first step to verifying the intramolecular interactions suggested by comparative patch analysis. The two functional states hypothesis, outlined in the Discussion, points to a number of experiments that could shed light on the structure and function of PSD-95. First, the proposed regulation of the PSD-95 activity by PDZ_3_–specific C-terminal peptides can be further tested using immunoprecipitation and yeast two-hybrid experiments similar to those performed for other GK-mediated interactions [60] (e.g., with the GKAP protein [57]). If the proposed regulation mechanism is verified, experimental control of the PSD-95 activity may become possible, enabling detailed study of the functional differences between the two states. Next, the intramolecular interactions proposed here can be tested by a variety of experimental techniques [64], including NMR spectroscopy [65], site-directed mutagenesis [66], hydrogen/deuterium exchange combined with mass spectrometry [67], and small angle X-ray scattering (SAXS) [68]. In particular, site-directed mutagenesis [66] of the interface residues in the first proposed state (see Datasets S1 and S2) could be used with pull-down assays to validate the predicted interaction interface [69]. In addition, the lack of accessibility of the GBS in the second state could be tested using nucleotide-binding assays [70,71]. Finally, the shapes of the calculated SAXS spectra for the best-scoring models in both conformations are substantially different (Figure 3). Thus, we expect the experimentally obtained SAXS spectra to be helpful in distinguishing between the two PSD-95 states.

Comparative patch analysis for characterizing the quaternary structure of protein assemblies provides a framework for combining data from known protein structures with a physical assessment of protein interactions. This framework will benefit from future developments in protein–protein docking, such as the explicit treatment of flexibility and more accurate scoring functions. We are currently developing an automated comparative patch analysis pipeline for large-scale modeling of protein complexes via a Web server. In closing, we expect that comparative patch analysis will provide useful spatial restraints for the structural characterization of an increasing number of binary and higher order protein complexes, as it did for PSD-95.

## Materials and Methods

### Comparative patch analysis protocol.

We start by outlining the steps in comparative patch analysis, followed by a more detailed description. First, for each partner domain in a binary complex, a set of protein binding sites of its homologs represented in PIBASE was identified [72]. Second, these binding sites were mapped onto the partner domain surface using structure-based alignments between the domain and each of its homologs. Third, all pairs of the mapped binding sites were converted by restrained docking to obtain candidate models of the binary complex. This ensemble of models was then ranked using a measure of geometric complementarity and a statistical potential score.

*Extracting and mapping binding sites of domain homologs.* For each of the two partner domains, we first defined a family of its homologs. Several schemes both dissect proteins into domains and cluster them into families, based on sequence, structure, and/or function [73–76]. We used the family definitions in SCOP [73]. Domains that belong to the same SCOP family usually share at least 30% sequence identity or the same biological function.

For a given SCOP family, the set of binary domain interfaces between its members and other domains was obtained from PIBASE, our comprehensive relational database of all structurally characterized interfaces between pairs of protein domains [72]. The domain–domain interfaces in PIBASE were extracted from protein structures in the Protein Data Bank (PDB) [77] and Protein Quaternary Structure (PQS) server [78] using domain definitions from the SCOP and CATH domain classification systems [73,74]. An interface is defined by a list of pairs of residues, one from each protein, that are in contact with each other. Each binding site consists of the residues that are within 6.05 Å of its partner domain, where the threshold is defined between any two nonhydrogen atoms.

The binding site residues from all domain family members were then mapped onto the partner domain using structure-based alignments obtained by DaliLite. DaliLite uses a Monte Carlo procedure to find the best alignment by optimizing a similarity score defined in terms of equivalent intramolecular distances [79].

*Modeling protein complexes.* The structures of binary protein complexes were predicted by restrained docking using the PatchDock software [80,81]. PatchDock uses an algorithm for rigid body docking that searches for the maximal geometric complementarity between two protein structures, optionally restrained by having to match two user-specified binding sites. Here, we provided all pairs of mapped binding sites, one from each target domain, as input for individual PatchDock runs. When a resulting refined model was inconsistent with the specified binding sites, it was discarded. More specifically, a model was considered not to correspond to a specified binding site interaction if the binding sites predicted by docking had less than 75% of their residues in common with the specified binding sites (the normalization is based on the size of the smaller of the specified and predicted binding sites).

The resulting binary complexes were scored using a combination of two independent scores, the geometric complementarity function of PatchDock and DOPE (Discrete Optimized Protein Energy) score. DOPE is a distance-dependent pairwise statistical potential calculated from known protein structures and available through the MODELLER program [82,83]. The configurations in the ensemble of models were ranked by a sum of the PatchDock and DOPE scores, first scaled to lie in the range between 0 and 1.

### Assessment of comparative patch analysis.

A benchmark set of 20 binary domain complexes was used to evaluate comparative patch analysis (Table 1). These complexes were divided into two groups. Each subunit of a complex in the first group is a member of a SCOP family that has been observed to interact with only one other SCOP family. In contrast, each subunit from the second group of complexes comes from a SCOP family that has been observed to interact with multiple SCOP families. The complexes were randomly selected from PIBASE such that the number of interactions available for the families of each component ranged between ten and 100. In total, there are 11 protein complexes (noncovalently linked domains) and nine multidomain proteins (covalently linked domains) in the benchmark set.

As in previous data-dependent approaches for modeling the structures of protein interactions [18,84,85], we have tested our method using a benchmark set designed within its scope of applicability. Our method is applicable only to protein complexes for which structures of the subunits or their homologs interacting with other proteins are available. This constraint on applicability also applies to the benchmark structures used to test the method. For this reason, we did not use the two benchmark sets that are generally used for protein docking methods, the set of CAPRI targets [16,86] and a benchmark set developed by Weng and coworkers [87]. The set of 19 CAPRI targets, whose structures are publicly available, was not an appropriate benchmark for our method because the majority of the structures either (i) contain subunits consisting of multiple SCOP domains (*n* = 7: T02–T07, T19), (ii) are not annotated by SCOP (*n* = 4: T09, T13, T20, T21), or (iii) there are no observed binding sites available for patch analysis (*n* = 4: T11, T12, T15, T19). This leaves five structures (T01, T08, T10, T14, T18) on which comparative patch analysis can be tested. Similarly, of the 63 rigid-body docking targets in the Weng benchmark set, 37 contain subunits with multiple SCOP domains and two contain subunits for which there are no observed binding sites available for comparative patch analysis. The remaining 24 targets contain subunits for which there is an average of 850 binding sites available for our method. This number of binding sites makes comparative patch analysis computationally very expensive, requiring on average more than two million localized docking calculations per target. There are only five targets in the Weng set that require no more than ten thousand calculations, the threshold we used in selecting our benchmark set. We are currently developing a method to cluster binding sites that would allow a significant reduction in the number of docking calculations required for a target structure, enabling the use of a more comprehensive benchmark set.

Adapting existing benchmarks to assess our method required ad hoc processing such as assigning domain boundaries and classifications, dissecting multidomain complexes into binary domain interactions, and reducing the number of input binding sites. Instead, we developed a benchmark set that is applicable to our method in an automated fashion. In addition, our benchmark set was designed to assess the performance of comparative patch analysis for domain–domain interactions in both multidomain proteins and protein complexes. The targets in the CAPRI and Weng benchmark sets are exclusively protein–protein interaction structures.

To quantify the amount of additional information provided by comparative patch analysis relative to docking, the structure of each protein complex was modeled using three independent protocols, relying on the docking program PatchDock (Methods). In the first protocol, known binding sites for the homologs of both subunits were used to restrain the docking. In the second protocol, known binding sites for the homologs of only one subunit were used to restrain the docking. In the final protocol, no binding site information was used, and conventional protein docking was applied.

*Distance metrics.* To evaluate the accuracy of comparative patch analysis in predicting the interaction interface and relative orientation of two structurally defined protein domains, the following three measures were used: binding site overlap, interface overlap, and RMS error.

First, we calculate the binding site overlap (*O_B_*), which we define as the percentage of correctly predicted binding site residues:

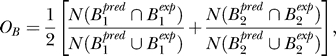
where 
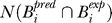
 is the number of residues in common between the predicted and actual binding sites, and 
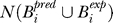
 is the total number of contact residues in both binding sites.

Next, we used the interface overlap (*O_I_*), as a measure to assess the predicted interface between the binding sites:

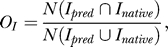
where 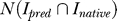 is the number of residue contacts in common between the predicted (*I_pred_*) and native (*I_native_*) interfaces, and 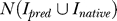 is the total number of residue contacts. Interfaces were deemed to be correct when at least half of the residue contacts were identified.

Finally, we calculated the all-atom RMS error between the predicted and native complexes using the L_RMS measure defined in CAPRI [88]. The predicted and native structures were superposed using the larger of the two domains, and the RMS error was calculated for the other domain.

### Modeling the PDZ_3_–SH3–GK complex of rat PSD-95.

*Comparative patch analysis application.* Comparative patch analysis was used to predict the tertiary structure of the rat PSD-95 core fragment that contains the PDZ_3_, SH3, and GK domains. From PIBASE, 126, 298, and 517 protein binding sites were obtained for the PDZ_3_, SH3, and GK domains, respectively. The binding sites were mapped onto the target structures. Redundant binding sites were removed so that no pair of binding sites shared more than 95% of their residues, leaving 49, 26, and 219 binding sites for the PDZ_3_, SH3, and GK, respectively. The comparative patch analysis protocol was then applied.

We then assessed whether the models were compatible with the 14-residue linker length between the PDZ_3_ and SH3 domains. To do so, the linker was modeled as a flexible chain of 14 spheres with 1.9 Å radii and a maximum distance of 3.8 A between consecutive spheres, to mimic the excluded volume of the linker and restrict the maximum spatial separation of the domains. Each model was assessed using the following protocol in MODELLER [83]. First, the positions of the 14 linker residues were placed at random coordinates and then optimized using simulated annealing molecular dynamics and conjugate gradient minimizations. The scoring function consists of terms equal to 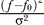 , where *f* is the restrained distance and *σ* is the parameter that regulates the strength of the term. Linker distances are restrained if *f* > *f*_0_, where *f*_0_ = 3.8 and *σ* = 0.05. Excluded volume restraints between the protein and the linker are imposed if *f* > *f*_0_, where *f*_0_ is the sum of the atomic and linker radii and *σ* = 0.01. The optimization of the scoring function was performed in 20 independent trials for each model, and the optimized coordinates of the linker residues with the lowest score were added to the model. As a result of assessment, those models that violated the imposed linker restraints and thus could not have an interdomain linker of such length between PDZ_3_ and SH3 domains were removed from the ensemble.

*Exhaustive docking.* The PDZ_3_–SH3–GK models built by comparative patch analysis were compared with those built by exhaustive docking using PatchDock without prior information about the potential binding site [80,81]. The model with the best PatchDock–DOPE score that satisfied the interdomain linker restraint was selected.

*Sequence analysis.* The SMART domain annotation tool was used to search for proteins containing the PDZ, SH3, and GK domains [89,90]. Proteins and splice variants annotated as PSD-95 proteins were obtained from the UniProt sequence database [91]. The sequences were scanned for known SH3 binding motifs (PXXP, PXXDY, RXXK [47]) using grep regular expression search.

### Proteolysis of PSD-95.

Rat PSD-95 was cloned into pET47b (+) and expressed as a His-tagged fusion protein (∼83.4 kDa) in BL21 (DE3) pLysS cells at 37 °C. Cells were harvested 3–3.5 h after induction by 0.4 mM IPTG. The cell lysate was centrifuged at 17K RPM, and the supernatant was loaded onto a nickel NTA column (Qiagen, http://www1.qiagen.com) and eluted with an imidazole gradient (20 mM to 500 mM). The purest fractions were exchanged (using PD10 columns, Amersham Biosciences, http://www.amersham.com/) to: 20 mM Tris (pH 8), 150 mM NaCl, 5 mM DTT, 10% glycerol for limited proteolysis (protocol based on that of Stroh et al*.* [92]). Digests of PSD-95 were initiated by adding protease to the following final concentrations: 0.83 μg/ml sequencing grade modified Trypsin (Roche, http://www.amersham.com), 0.1 μg/ml of proteinase (Fluka, http://www.sigmaaldrich.com), or 8.3 μg/ml of thermolysin (Sigma, http://www.sigmaaldrich.com). The thermolysin reaction was also supplemented with 5 mM CaCl_2_. Digests were incubated at 37 °C and stopped with 5 mM PMSF for trypsin and proteinase, and 10 mM EDTA for thermolysin. Aliquots were taken at 5, 30, 60, 90, 120 min, and 8 h after addition of protease and flash frozen in liquid nitrogen until analysis by SDS-PAGE. Stable fragments were excised from Coomassie-stained gels and subjected to tryptic digestion in the gel piece after reduction with DTT and alkylation with iodoacetamide [92,93]. The tryptic peptides were extracted from gel slices with 5% formic acid in 50% acetonitrile, concentrated in a SpeedVac (Savant Instruments, http://www.combichemlab.com), and desalted with the use of a Zip Tip (Millipore, http://www.millipore.com) before analysis by MALDI–TOF (matrix-assisted laser desorption ionization–time of flight) mass spectrometry. Samples were mixed with either α-cyanohydroxycinnamic acid or a “Universal” MALDI matrix from Fluka. Analyses were performed with a Voyager DE-PRO MALDI–TOF mass spectrometer (Applied Biosystems, http://www.appliedbiosystems.com) that was first externally calibrated using a calibration mix supplied by the manufacturer. The MALDI spectra were recalibrated internally with known peptide masses, e.g., trypsin autolysis peaks or expected masses obtained from in silico digests of the known protein. The software, Prospector MSFIT (University of California San Francisco), was used to identify the tryptic fragments.

The representations of proteins in Figures 1–3 were obtained using Chimera [94], and in Figure 4 with the help of MolMol software [95].

## Supporting Information

Dataset S1 The Best Model of the First Configuration of PSD-95 Core Fragment (229 KB TXT)

Dataset S2 The Best Model of the Second Configuration of PSD-95 Core Fragment (229 KB TXT)

### Accession Numbers

Accession numbers from the Protein Data Bank (http://rcsb.org) for the proteins mentioned in this paper are: rat PSD-95 GK (1JXM), rat PSD-95 PDZ_3_ (1BE9), rat PSD-95 PDZ_3_ (1BFE), rat PSD-95 SH3 and GK (1JXO), pyruvate formate–lyase protein complex (1CM5).

Accession numbers from the European Bioinformatics Institute SMART database (http://www.ebi.ac.uk/interpro/) for proteins mentioned in this paper are: PSD-95 PDZ (SM00228), PSD-95 SH3 (SM00326), and PSD-05 GK (SM00072).

## Abbreviations

DOPE: Discrete Optimized Protein Energy
GBS: GMP-binding site
GK: guanylate kinase–like
MALDI–TOF: matrix-assisted laser desorption ionization–time of flight)
PDZ: PSD-95, DLG, and ZO-1
PDB: Protein Data Bank
PRBS: proline-rich binding site
PSD: postsynaptic density
SAXS: small angle X-ray scattering
SCOP: Structural Classification of Proteins
SH3: Src homology 3
